# Identification of Glucose Metabolism-Related Genes in the Progression from Nonalcoholic Fatty Liver Disease to Hepatocellular Carcinoma

**DOI:** 10.1155/2022/8566342

**Published:** 2022-11-03

**Authors:** Siyuan Wang, Yiling Li, Ning Liu, Wei Shen, Wenhao Xu, Peng Yao

**Affiliations:** ^1^Department of Hepatobiliary Surgery, Xiaogan Hospital Affiliated to Wuhan University of Science and Technology, Xiaogan 432000, China; ^2^Department of Anesthesiology, Xiaogan Hospital Affiliated to Wuhan University of Science and Technology, Xiaogan 432000, China; ^3^Department of Intensive Care Unit, Xiaogan Hospital Affiliated to Wuhan University of Science and Technology, Xiaogan 432000, China

## Abstract

Nonalcoholic fatty liver disease (NAFLD) is a manifestation of hepatic metabolic syndrome that varies in severity. Hepatocellular carcinoma progresses from NAFLD when there is heterogeneity in the infiltration of immune cells and molecules. A precise molecular classification of NAFLD remains lacking, allowing further exploration of the link between NAFLD and hepatocellular carcinoma. In this work, a weighted gene coexpression network analysis was used to identify two coexpression modules based on multiple omics data used to differentiate NAFLD subtypes. Additionally, key genes in the process of glucose metabolism and NAFLD were used to construct a prognostic model in a cohort of patients with hepatocellular carcinoma. Furthermore, the specific expression of signature genes in hepatocellular carcinoma cells was analyzed using a single-cell RNA sequencing approach. A total of 19 liver tissues of NAFLD patients were obtained from the GEO database, and 81 glucose metabolism-related genes were downloaded from the CTD database. In addition, based on nine signature genes, we constructed a prognostic model to divide the HCC cohort into high and low-risk groups. We also demonstrated a significant correlation between prognostic models and clinical phenotypes. Furthermore, we integrated single-cell RNA-sequencing data and immunology data to assess potential relationships between different molecular subtypes and hepatocellular carcinoma. Finally, our study discovered that the glucose metabolism pathway may play an important role in the process of NAFLD-hepatocellular carcinoma. In addition, three glucose metabolism-related genes (SERPINE1, VCAN, and TFPI2) may be the potential targets for the immunotherapy of patients with NAFLD-hepatocellular carcinoma.

## 1. Introduction

Globally, nonalcoholic fatty liver disease (NAFLD) affects approximately 25% of the adult population, making it the most common chronic liver disease [1]. As part of NAFLD, there are several types of liver disease, such as simple steatosis and nonalcoholic steatohepatitis with varying levels of fibrosis and even cirrhosis [2]. Since obesity and metabolic syndrome are becoming more prevalent, NAFLD has become the leading cause of abnormal liver enzymes in the United States [3]. About 25% of the world's population may suffer from NAFLD, which affects 1 billion people worldwide [4]. A substantial difference exists in the prevalence of NAFLD in different parts of the world. NAFLD prevalence is highest in the Middle East and South America and lowest in Africa [5]. As many as 80 million people in the U.S. may have NAFLD. An individual with 5% hepatocyte infiltration with steatosis is considered to have NAFLD when they undergo imaging or liver biopsy testing [6]. The majority of people with NAFLD are asymptomatic, and they may remain silent until they develop cirrhosis [7]. Patients with NAFLD often suffer from fatigue and pain in the right upper quadrant when they are initially referred. Individuals with NAFLD may have an echogenic liver on ultrasound or evidence of liver fat on imaging studies [8]. Cardiovascular disease is the leading cause of death among NAFLD patients, followed by cancer and liver disease [9]. The adjusted hazard ratio for cardiovascular disease in nonobese people with NAFLD was approximately 10 times higher than in individuals without NAFLD in a Japanese cohort [10].

Glucose metabolism in the liver is critical to protein and lipid glycosylation. Diabetes and other chronic diseases may have metabolic changes due to alterations in glucose metabolism in the human liver. Understanding the glucose metabolism pathways in the healthy liver may help to shed light on these changes [11]. It is believed that NAFLD results from the imbalance in the hepatic energy metabolism, where excessive energy enters the liver relative to its ability to oxidize it into carbon dioxide or very low-density lipoprotein [12]. Therefore, energy is accumulated in the liver in the form of triglycerides, which may explain the common occurrence of NAFLD in obese and lipodystrophic patients [13]. Although excessive consumption of any food can lead to the development of NAFLD, monosaccharides and disaccharides, especially fructose, sucrose, and high fructose corn syrup, which are prevalent in processed foods, can further exacerbate NAFLD by activating de novo lipogenesis programs in the liver [14]. Moreover, fructose is almost entirely metabolized by the liver, and dietary fructose is converted into triglycerides by de novo lipogenesis [15].

NAFLD has become the leading cause of hepatocellular carcinoma and end-stage liver disease in the past decade. It is now well established that hepatocellular carcinoma can develop in NAFLD without cirrhosis, even though it has previously been considered the end stage of liver disease progression [16]. It is estimated that liver cancer cells consume an enormous amount of energy during proliferation and escape from apoptosis [17]. Glucose metabolism and fatty acid oxidation are altered to support proliferation and escape apoptosis [18]. It is also possible that altered glucose metabolism can result in elevated levels of saturated and monounsaturated fatty acids, which may prevent oxidative damage to cancer cells [19].

## 2. Methods

### 2.1. Datasets Downloaded

Genome-wide analysis of gene expression in NAFLD patients and healthy livers is downloaded from GSE89632 (https://www.ncbi.nlm.nih.gov/, GEO). In addition, glucose metabolism-related genes were downloaded from the Comparative Toxicogenomics Database (CTD, https://ctdbase.org/). The gene expression data as well as the clinical information of hepatocellular carcinoma patients were downloaded from the Cancer Genome Atlas database (https://portal.gdc.cancer.gov/, TCGA). Single-cell RNA expression data from multiregional sampling in hepatocellular carcinoma were downloaded from GSE112271 in the GEO database.

### 2.2. Exploration of the Differential Expression Genes

Data on gene expression were obtained from the TCGA and GEO databases, and differential expression of mRNA was investigated using the Limma package in R. An adjusted *P* value of 0.05 in TCGA or GEO was defined as a threshold to distinguish between mRNAs, while |log_2_(fold change) | > 1 was defined as a threshold for mRNA differential expression screening. A gene annotation tool, the Gene Ontology (GO), is widely used to annotate genes with functions, particularly molecular functions (MFs), biological pathways (BPs), and cellular components (CCs). A KEGG enrichment analysis can be effective for analyzing gene function and related genomic functional information at a high level. An analysis of the KEGG pathway enrichment and GO function of underlying mRNAs was conducted using the ClusterProfiler package in R to better understand the oncogenic potential of target genes.

### 2.3. Subtype of the Expression Data

A consistency analysis was performed using the package ConsensusClusterPlus (v1.54.0), and heatmaps for gene expression were generated using genes with a variance greater than 0.1. R is used to implement all the above analysis methods.

### 2.4. Timer Database Analysis

An analysis of the correlation between immune infiltrating cells and tumor immunity was performed with the TIMER module (https://cistrome.shinyapps.io/timer/). Additionally, we used CellMarker to search for immune gene markers (https://biocc.hrbmu.edu.cn/CellMarker/). The correlations between gene expression levels and markers for immune genes can be visualized using expression plots.

### 2.5. Construction of the Prognostic Prediction Model Based on Glucose Metabolism Related Genes

Data and clinical information on hepatocellular carcinoma are downloaded from the TCGA dataset repository (https://portal.gdc.com). After extracting the data in TPM format from it and normalizing it to log2(TPM + 1), we retained samples with RNAseq data and clinical information. A KM survival analysis was conducted using the log rank to determine whether there was a statistically significant difference between the groups above in terms of survival. For the prediction model's accuracy, a timeROC analysis was performed. The least absolute shrinkage and selection operator (LASSO) regression algorithm was used for feature selection, and 10-fold cross-validation was used. The log-rank test and univariate Cox regression were used for calculating *P*-values and hazard ratios (HR) with 95% confidence intervals (CI) for Kaplan–Meier curves. Statistical significance was defined as a *P* < 0.05 for all of the above analysis methods and R packages, which were performed using R software version 4.2.1.

### 2.6. Gene Set Enrichment Analysis (GSEA)

MSigDB was used to retrieve gene sets. GSEA was performed on the gene sets to identify enriched GO terms and KEGG pathways. The 50 best terms were selected from each subtype based on their significance.

### 2.7. Immune Scores, Immune Checkpoints, and Immunotherapy Responses

In order to explore the immune scores, we used immunedeconv, which is an R package integrating six state-of-the-art algorithms, including TIMER, xCell, MCP-counter, CIBERSORT, EPIC, and quanTIseq. Based on the TCGA dataset, we obtained clinical information about patients with hepatocellular carcinoma. SIGLEC15, TIGIT, CD274, HAVCR2, PDCD1, CTLA4, LAG3, and PDCD1LG2 are genes related to immune checkpoints, and the expression of genes related to immune checkpoints was evaluated in R. In addition, the TIDE algorithm is used to predict possible immunotherapy responses.

### 2.8. Preprocessing and Quality Control of Single Cell RNA-Seq Data (10× Genomics)

The single-cell RNA-seq dataset was derived from the GEO database's supplementary file. In addition to filtering out poor-quality cells using the Seurat package, standard data preprocessing pipelines were used to generate the objects. Genes with fewer than three cells detected were filtered, as were genes with fewer than 200 genes detected. A minimum of 10,000 cells were used in the analysis, and cells with fewer than 200 or more than 2,500 genes detected, as well as cells with a high mitochondrial content, were filtered out. By adjusting the scale factor to 10,000, we normalized each cell. The ScaleData function from Seurat is used to normalize the data after it has been log-transformed. A normalized set of data measures was applied to standard analyses, as described in the Seurat R package. In UMAP, the first 30 principal components are used for visualization and clustering. A cell clustering procedure was performed using the FindClusters function (resolution = 0.2) in the Seurat R package.

## 3. Results

### 3.1. Identification of the DEGs in the NAFLD Cohort and the Glucose Metabolism-Related Genes

**Figure 1:**
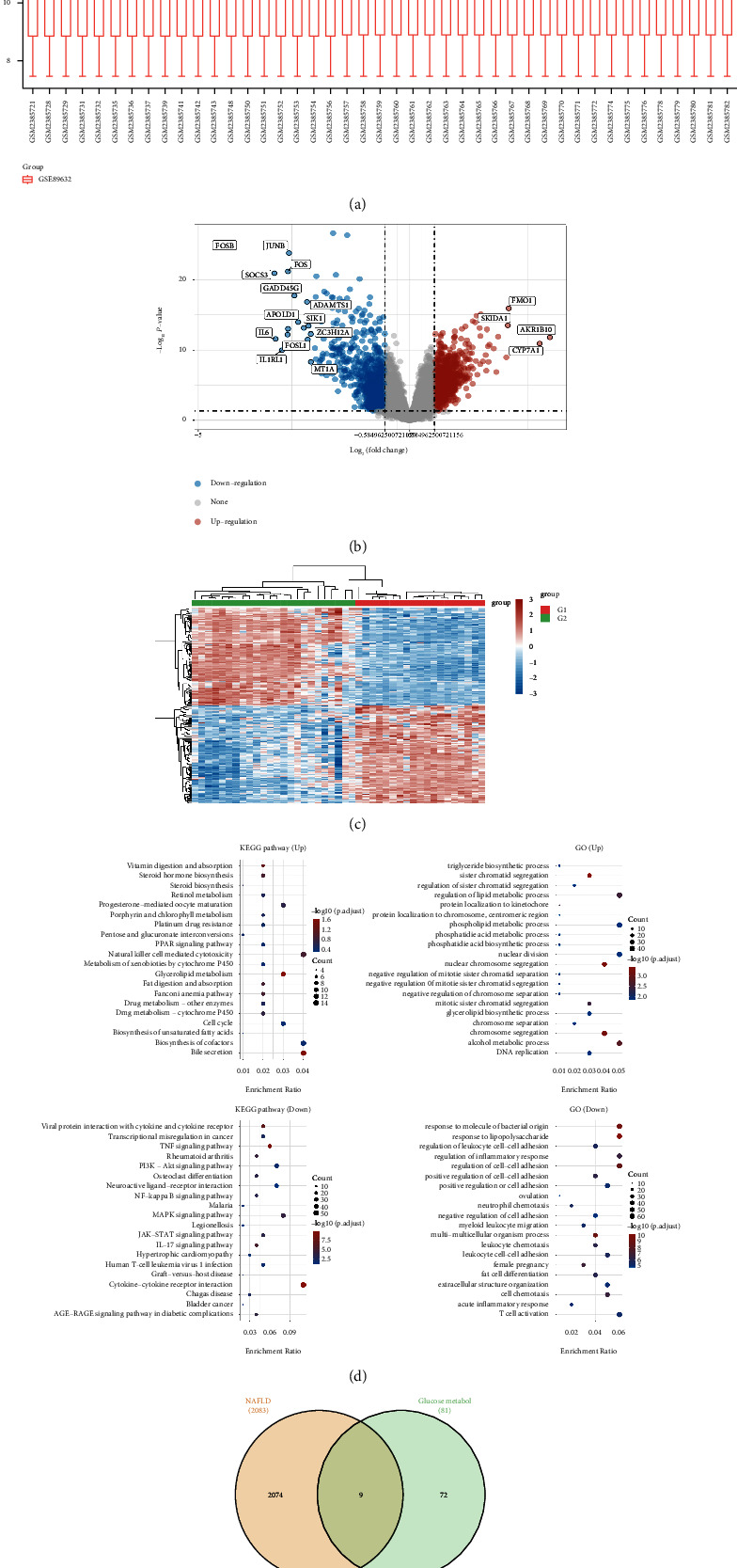
(a) Data normalization boxplots. (b) In this differential gene volcano plot, the red dots represent significantly differentially upregulated genes, and the blue dots represent significantly differentially downregulated genes. (c) The heatmap shows the differential expression of genes across different tissues in terms of colors. (d) A functional enrichment analysis of differentially expressed genes based on KEGG and GO pathways. (e) The Venn diagram shows the genes that are associated with NAFLD and glucose metabolism.

A total of 24 normal liver tissues and 19 liver tissues of NAFLD patients were involved in the GSE89632 cohort (Figure 1(a)). The differential expression analysis between NAFLD patients and control groups was performed in R. The results demonstrated that 925 genes were upregulated and 1158 genes were downregulated in the NAFLD patients compared with normal people (Figures 1(b)-1(c)). The GO and KEGG enrichment analysis revealed that many pathways were closely correlated with NAFLD (Figure 1(d)). In addition, in order to explore the role of glucose metabolism in the NAFLD patients, we then obtained a total of 81 glucose metabolism-related genes were downloaded from the CTD database. The Venn diagram demonstrated that 9 key genes are involved in both the NAFLD and glucose metabolism pathways, including GCK, PPP1R3C, NHLRC1, ENO3, PPP2R5D, PFKFB3, PGM2, SLC25A12, and PFKP (Figure 1(e)).

### 3.2. Exploration the Role of Key of Immune-Related Genes in the NAFLD Cohort

**Figure 2:**
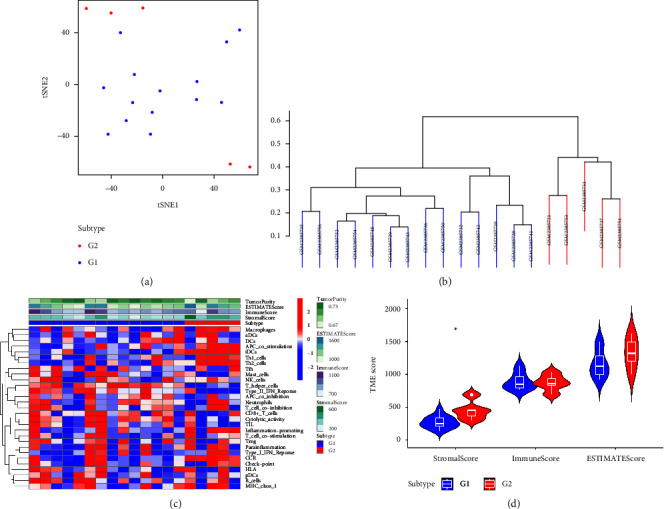
(a)-(b) The subtype based on the expression of immune-related genes. (c) The different expression of immune cells between different subtypes. (d) The different immune scores between different subtypes.

Subsequently, based on the expression level of immune-related genes, the expression data of the NAFLD cohort were divided into high- and low-immune score groups (Figures 2(a)-2(b)). The results revealed that the immune cells were differentially expressed between the G1 and G2 groups. In addition, the G2 group shows a higher stromal score compared with the G1 group (Figure 2(c)). While the immune score and estimate score show no difference between the G1 and G2 groups (Figure 2(d)).

### 3.3. The Subtype Based on 9 Key Genes Was Closely Associated with the Prognosis of Hepatocellular Carcinoma Patients

**Figure 3:**
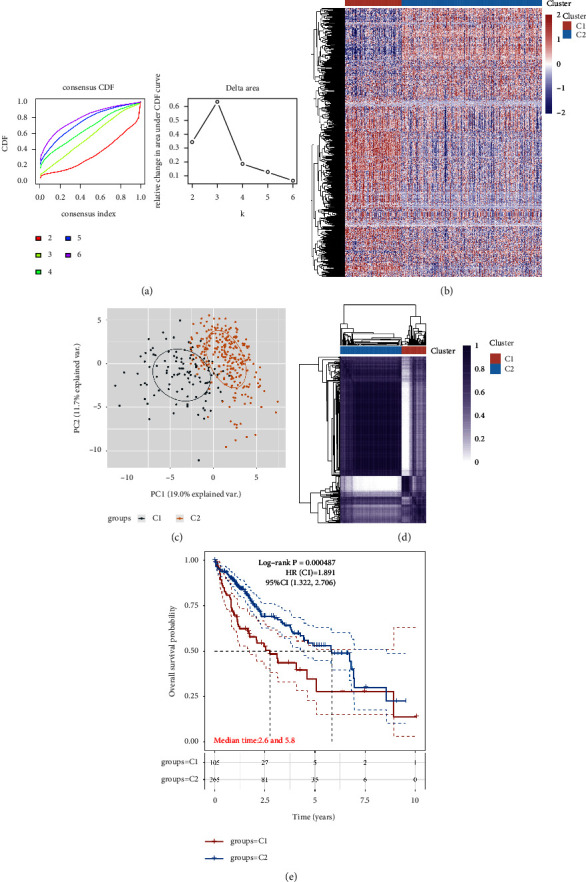
(a) Typed CDF curve of hepatocellular carcinoma cohort. (b) Typed CDF Delta area curve of hepatocellular carcinoma cohort. (c) Plot of PCA results after batch removal. (d) Heatmap of ConsensusClusterPlus consistent clustering results when *k* = 2. (e) Different subgroup samples in the data set are represented by KM survival curves, in which log rank is used to test for different groups, and 95% CL represents the HR confidence interval for each group.

In order to explore the relationship between NAFLD and hepatocellular carcinoma and figure out the role of the glucose metabolism pathway in hepatocellular carcinoma induced by NAFLD, the patients involved in hepatocellular carcinoma were divided into C1 and C2 groups based on the expression level of 9 key genes. For concordance clustering, delta area curves indicate the change in the area under the cumulative distribution function (CDF) curve for each category number *k* compared to *k* − 1 (Figure 3(a)). The ConsensusClusterPlus consistent clustering heat map shows red for high expressions and blue for low expressions when *k* = 2 (Figures 3(b)-3(d)). There are significant differences between the overall survival rates of the C1 group and the C2 group according to the KM survival curves of different subgroup samples in the dataset. The results revealed that the glucose metabolism-related genes involved in NAFLD are closely associated with the prognosis of hepatocellular carcinoma patients (Figure 3(e)).

### 3.4. Construction of the Prognostic Prediction Model Based on Glucose Metabolism Related Genes Involved in NAFLD in the Hepatocellular Carcinoma Cohort

**Figure 4:**
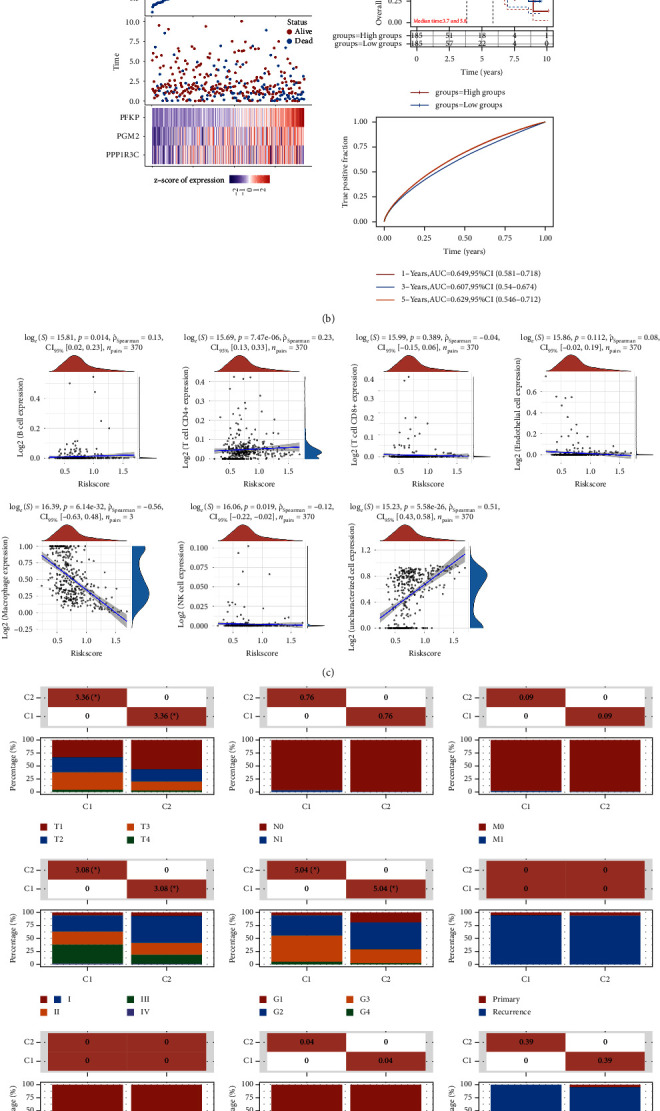
(a) We plotted partial likelihood deviations against log (*λ*) using a LASSO Cox regression model. (b) Based on log rank, the survival curve distribution of the risk model is tested for different groups. The risk model ROC curve and AUC values at different times, the higher the AUC value, the more accurate the model. (c) Spearman's correlation analysis between model scores and immune cell scores. (d) The correlation analysis between model scores and clinical information.

**Figure 5:**
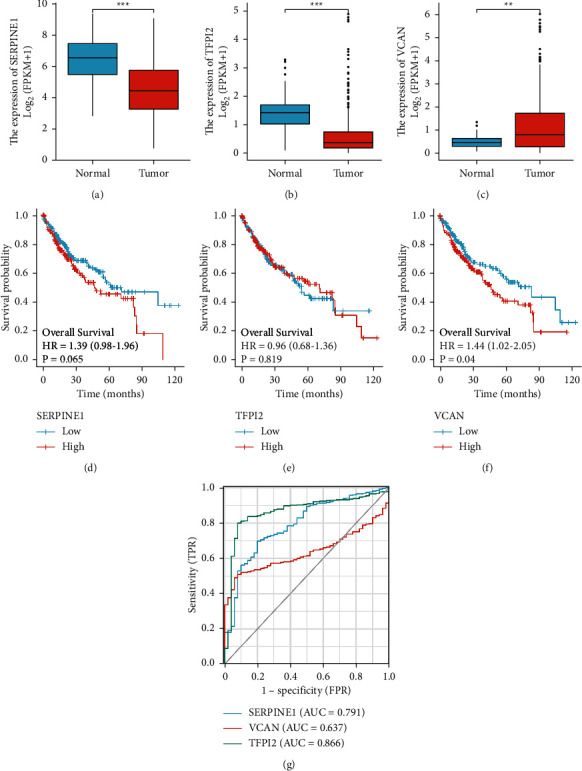
(a) Boxplots showing the differential expression of SERPINE1 in tumor patients and the normal population. (b) Boxplots showing the differential expression of TFPI2 in tumor patients and normal population. (c) Boxplots showing the differential expression of VCAN in tumor patients and the normal population. (d) The survival analysis demonstrated the low- and high-expression groups of SERPINE1. (e) The survival analysis demonstrated the low- and high-expression groups of TFPI2. (f) The survival analysis demonstrated the low- and high-expression groups of VCAN. (g) The ROC curve demonstrates the predictive value of SERPINE1, TFPI2, and VCAN.

**Figure 6:**
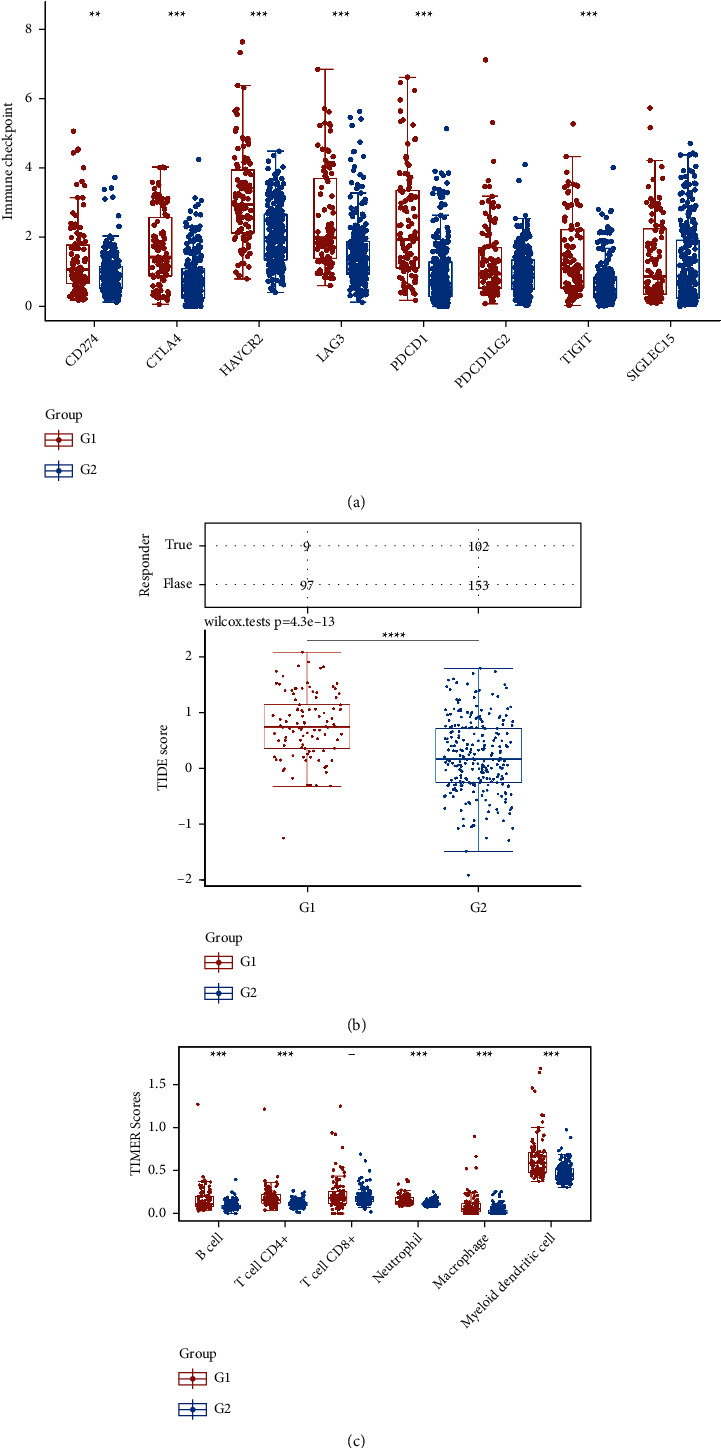
(a) Identifying immune checkpoint genes that are expressed differently in high-risk and low-risk populations. (b) Scores of immunotherapy response in high- and low-risk groups. (c) Spearman's correlation analysis between model scores and immune cell scores.

Subsequently, in order to further obtain the genes that are closely associated with the prognosis of hepatocellular carcinoma patients, we then performed the lasso regression analysis. The lasso regression analysis revealed that three glucose metabolism-related genes involved in NAFLD were applied to the prognosis prediction model (the risk score = (0.1177) × SERPINE1 + (0.0046) × VCAN + (0.0141) × TFPI2) (Figure 4(a)). Depending on the median risk score, patients were categorized as either low-risk or high-risk groups. In addition, the Kaplan–Meier curve showed that the prognostic model was closely related to the prognosis of hepatocellular carcinoma patients. Furthermore, the ROC curve results show that the AUCs are all greater than 0.6 at 1, 3, and 5 years, which indicates that the model is of good predictive value (Figure 4(b)). The expression level of B cells and CD4^+^ T cells is positively correlated with the risk score. In addition, the expression levels of endothelial cells, macrophages, and NK cells were negatively correlated with the risk score (Figure 4(c)). The clinical correlation analysis revealed that the risk score is closely related to the T stage, stage, and grade of hepatocellular carcinoma patients (Figure 4(d)). We then evaluated the expression of SERPINE1, VCAN, and TFPI2 in the hepatocellular carcinoma cohort of the TCGA cohort. The results demonstrated that VCAN was downregulated in the hepatocellular carcinoma samples compared with normal samples (Figure 5(c)). While SERPINE1 and TFPI2 were upregulated in hepatocellular carcinoma samples compared with normal samples (Figures 5(a)-5(b)). The KM survival curve revealed that VCAN is associated with the prognosis of hepatocellular carcinoma patients (*P* < 0.05) (Figures 5(d)–5(f)). The time-dependent ROC curve showed that the AUC value for TFPI2, SERPINE1, and VCAN was 0.866, 0.791, and 0.637, respectively (Figure 5(g)). Our next step was to examine differences in immune checkpoint expression between the groups. A significant difference was observed between high- and low-risk groups in the expression of CD274, CTLA4, HAVCR, LAG3, PDCD1, and TIGIT, which may be the potential targets for immunotherapy (Figure 6(a)). An assessment of tumor immune escape mechanisms was conducted using the TIDE score. According to the TIDE score results, the low-risk group received immune checkpoint blockade therapy with low efficacy, indicating that they received an immune checkpoint blockade therapy that was not effective (Figure 6(b)). According to the immune cell scores, high-risk and low-risk groups had significantly different scores for B cells, CD4+ T cells, neutrophils, macrophages, and myeloid dendritic cells (Figure 6(c)).

### 3.5. Single-Cell RNA Seq Defines Key Gene Expression Heterogeneity in Hepatocellular Carcinoma

**Figure 7:**
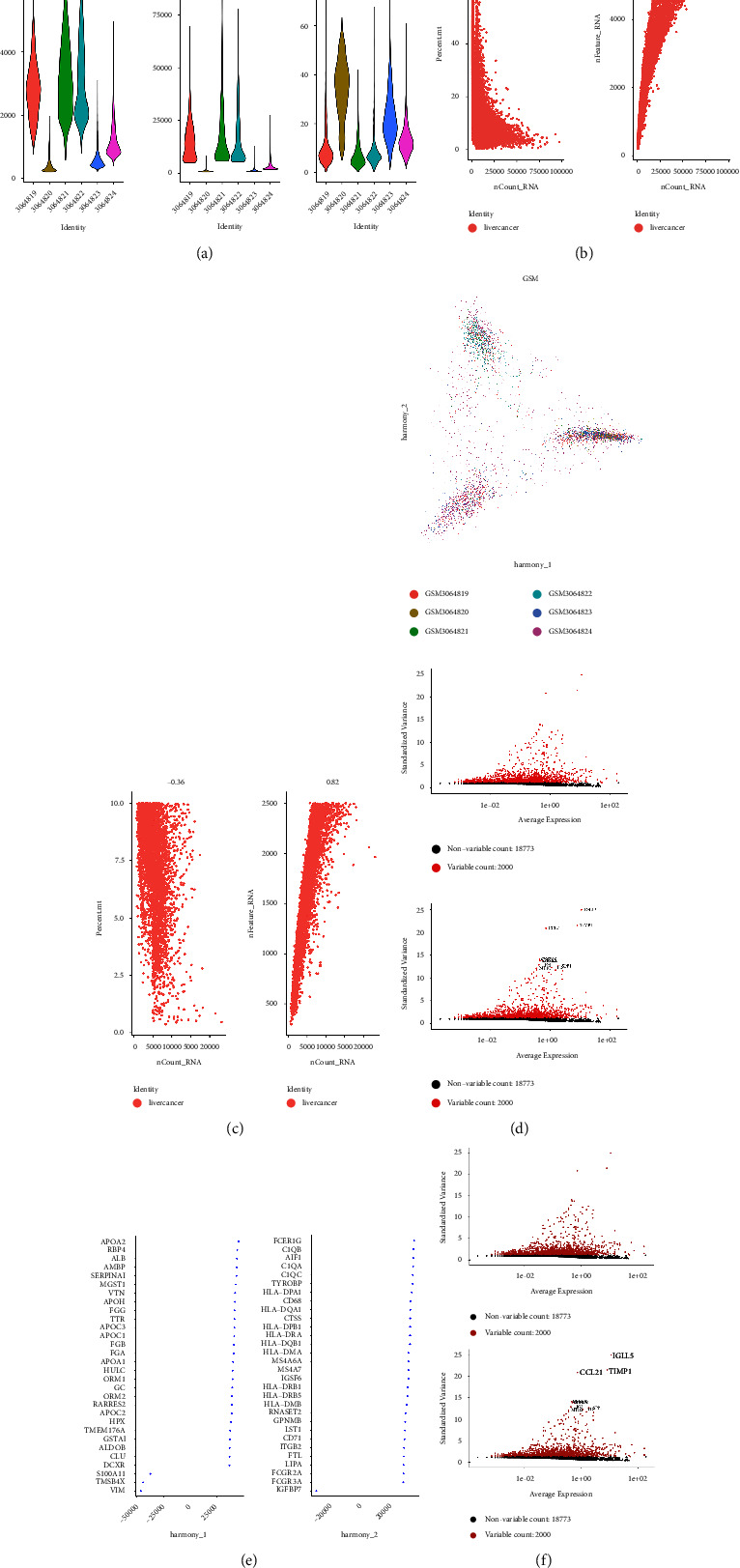
(a) The amount of mitochondrial genome detected in each cell, the number of molecules detected in the cell, and the number of genes detected in each cell. (b) Comparison of underlying data before filtering. (c) The correlation between filtered data and the underlying data. (d) Using PCA analysis, multiple PC populations with significant differences can be used as anchor points, showing two parts of PC1 and PC2. (e)-(f) Each point represents a gene, and red represents the top 10 hypervariable genes after batch removal.

**Figure 8:**
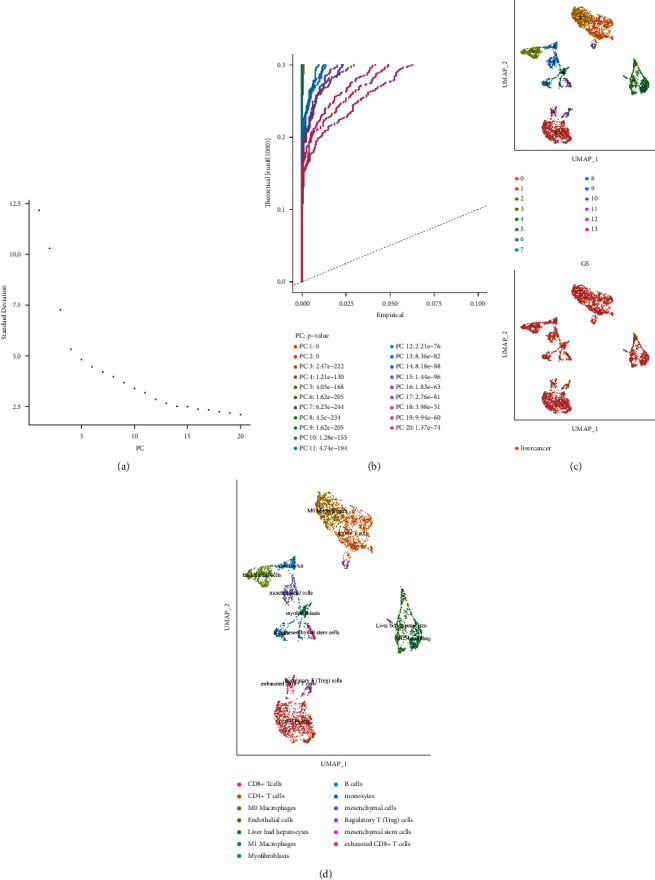
(a) The ElbowPlot function is used to evaluate PCs. (b) A visual representation of JackStrawPlot, which compares *P*-value distributions for PCs to a uniform distribution. (c) Cell groups are represented by different numbers or colors when using UMAP or TSNE methods. (d) Cell populations are represented on the map by different numbers or colors after they have been manually annotated.

**Figure 9:**
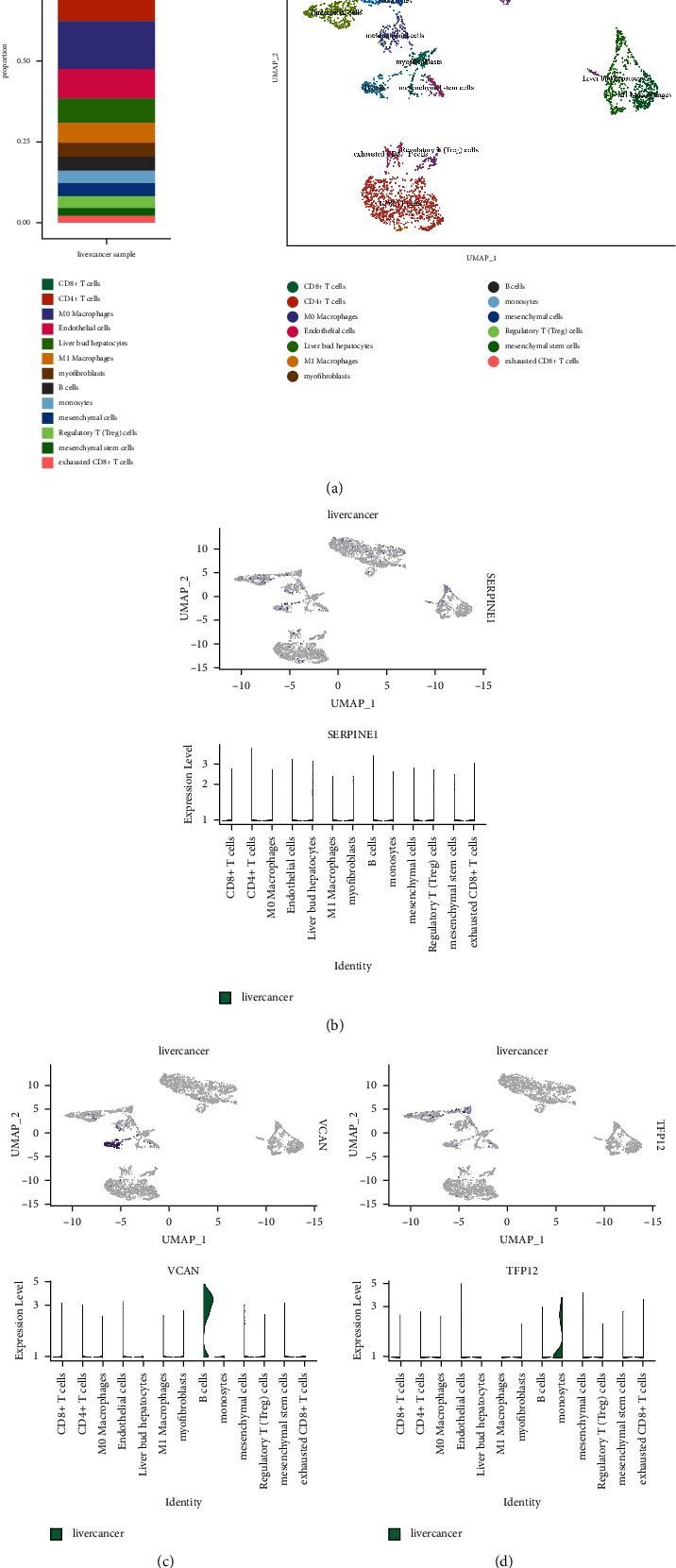
(a) Different groups' distributions of cell proportions. (b) Expression map of SERPINE1 in different groups. (c) Expression map of VCAN in different groups. (d) Expression map of TFPI2 in different groups.

A total of 6 samples from patients with hepatocellular carcinoma were involved in this study. A description of quality control can be found in materials and methods. Following the removal of batch effects and the regressing of unique molecular identifier (UMI) numbers and mitochondrial UMI counts, 27,350 cells passed quality control (Figure 7). These cells are grouped into 13 major cell lineages, including CD8+ T cells, CD4+ T cells, M0 macrophages, endothelial cells, liver bud hepatocytes, M1 macrophages, myofibroblasts, B cells, monocytes, mesenchymal cells, Treg, mesenchymal stem cells, and exhausted CD8+ T cells (Figure 8).  Figure 9 shows the distribution of cell proportions in different groups. Then, we evaluated the expression level of SERPINE1, VCAN, and TFPI2 in human hepatocellular carcinoma cells. The results demonstrated that SERPINE1 is rarely expressed in hepatocellular carcinoma cells. VCAN is specifically expressed in B cells of hepatocellular carcinoma. In addition, TFPI2 is specifically expressed in the monocytes of hepatocellular carcinoma.

### 3.6. Exploration of the Potential Function of SERPINE1, VCAN, and TFPI2 in the Hepatocellular Carcinoma Cohort

**Figure 10:**
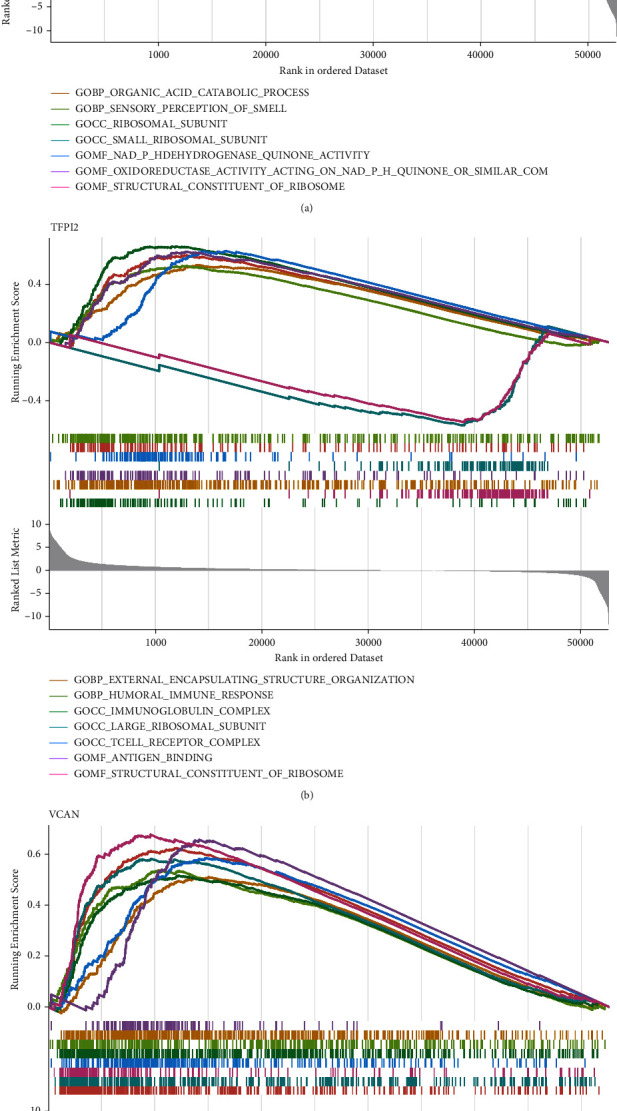
(a) GSEA enrichment analysis of the SERPINE1 expression. (b) GSEA enrichment analysis of the TFPI2 expression. (c) GSEA enrichment analysis of the VCAN expression.

Finally, in order to explore the function of 3 key genes (SERPINE1, VCAN, and TFPI2) in hepatocellular carcinoma patients, we then performed the GSVA enrichment analysis. The results revealed that SERPINE1 is mainly enriched in a structural constituent of ribosome, ribosomal subunit, sensory perception of smell, organic acid catabolic process, and oxidative phosphorylation (Figure 10(a)). For VCAN, the GSEA enrichment analysis demonstrated that VCAN is closely associated with external encapsulating structure organization, collagen-containing extracellular matrix, extracellular matrix structural constituent, plasma membrane signaling receptor complex, skeletal system development, T cell receptor complex, and immune response regulating signaling pathway (Figure 10(c)). In terms of TFPI2, the results of GSEA enrichment analysis revealed that many pathways are involved in TFPI2, including immunoglobulin complex, structural constituent of ribosome, external encapsulating structure organization, antigen binding, large ribosomal subunit, T cell receptor complex, complement activation, humoral immune response, and ribosomal subunit (Figure 10(b)).

## 4. Discussion

Approximately 25% of the world's adult population suffers from NAFLD, which is the most common chronic liver disease [20]. The prevalence of NAFLD has been found to increase with age and may even lead to cirrhosis or hepatocellular carcinoma in some studies [21]. Individuals maintain health by maintaining glucose homeostasis in order to meet the energy requirements of vital organs [22]. In addition to glycogenesis, glycogenolysis, glycolysis, and gluconeogenesis, the liver plays a vital role in controlling glucose homeostasis [23]. However, few studies focused on the role of glucose metabolism in hepatocellular carcinoma induced by NAFLD. In this work, we first explore the genes that are closely related to NAFLD and glucose metabolism. The results revealed that a total of 9 genes were closely correlated with NAFLD and glucose metabolism, including GCK, PPP1R3C, NHLRC1, ENO3, PPP2R5D, PFKFB3, PGM2, SLC25A12, and PFKP.

The underlying problem with NAFLD is insulin resistance, a key factor in metabolic syndrome, which is also linked to type 2 diabetes and hypertriglyceridemia [24]. Patients with obesity may be at risk for NAFLD due to abnormal lipid and glucose metabolism [25]. Currently, most basic research appears to focus on insulin resistance as well as the failure of the liver to process glucose loads from a pathophysiological perspective [26]. A former study has discovered that the JKW modulates insulin signaling and glucose metabolism to alleviate NAFLD [27]. In addition, this study identifies scientific evidence supporting the potential efficacy of JKW for the prevention and treatment of NAFLD [28].

In order to further explore the role of glucose metabolism in hepatocellular carcinoma induced by NAFLD, we then constructed a prognostic prediction model based on 9 key genes. We finally discovered that SERPINE1, VCAN, and TFPI2 play an important role in hepatocellular carcinoma.

Recent studies have discovered that SERPINE1, VCAN, and TFPI2 are associated with many human tumors. The former study revealed that sh-TARBP2 cells with miR-145 overexpression were rescued from SERPINE1 inhibition and functional hepatoma cells were restored, which could be an important new intervention target in aggressive hepatocellular carcinoma. Many studies have found that VCAN may be a risk factor in gastric cancer, breast cancer, and colorectal cancer [29]. In addition, VCAN is a promising biomarker for the prognostic prediction of gastric cancer patients, breast cancer patients, and colorectal cancer patients [30]. Zhao et al.have discovered that TFPI2 inhibits breast cancer progression by inhibiting the TWIST-integrin pathways, presenting a new therapeutic target [31]. As a biomarker used in the colorectal cancer cohort, VCAN may assist in identifying patients at high risk for postoperative complications during stages II and III [32]. According to another study, TFPI2 gene methylation is an independent predictor of poor prognosis in nonsmall cell lung cancer patients [33]. In addition, our further research has revealed that 3 key genes are associated with immune checkpoint blockade therapy and immunotherapy of hepatocellular carcinoma, which may suggest that immunotherapy could be an effective way to treat hepatocellular carcinoma induced by NAFLD [34].

Previous studies have focused on the screening of differentially expressed biomarkers between tumor and nontumor tissues. It is possible to lose important genes when analyzing bulk transcriptome data from cell populations. Single cell-RNA sequencing analysis is therefore more useful in elucidating the underlying mechanisms of NAFLD and hepatocellular carcinoma. In this work, in order to explore the expression level of key genes in the different cells of hepatocellular carcinoma, we then performed single cell-RNA sequencing of hepatocellular carcinoma samples. The results demonstrated that VCAN is specifically expressed in B cells and is specifically expressed in monocytes. Liu et al. discovered that hepatocellular carcinoma is more responsive to immunotherapy by targeting monocyte-intrinsic enhancer reprogramming. Furthermore, an assessment of the lymphocyte-to-monocyte ratio predicts prognosis in hepatocellular carcinoma patients undergoing radiofrequency ablation and transcatheter arterial chemoembolization.

Additionally, we also evaluated the potential function of SERPINE1, VCAN, and TFPI2 in a hepatocellular carcinoma cohort. The results revealed that the humoral immune response is closely associated with TFPI2. According to growing evidence, the peripheral immune response to hepatocellular carcinoma affects how the disease develops, how it responds to therapy, and how long patients live. Furthermore, an immune-suppressive response was also found among patients with NAFLD-hepatocellular carcinoma, as determined by functional and metabolomic evidence [35]. An additional study demonstrated that AKR1B10 and SPP1 were closely related to NAFLD and NAFLD-hepatocellular carcinoma immune cell infiltration and immunosuppressive cytokine expression [36]. SERPINE1 is closely associated with immune checkpoint molecule expression in the GC cohort as a hypoxia-related gene [37]. In addition, there is a good correlation between VCAN and immune checkpoint blockade response [38].

In recent years, many studies have focused on the role of bioinformatics analysis methods in human health [39]. The bioinformatics analysis could lead to higher-quality research and provide new directions for researchers. However, there are also some limitations to bioinformatics analysis. First, without experimental verification, the results need to be verified by experiments [40]. In addition, high heterogeneity often leads to large bioinformatics analysis errors, so unifying the methods is essential to reducing errors [41]. Therefore, corresponding experimental validations are needed to be performed to further confirm the accuracy of our results.

Taken together, our study discovered that the glucose metabolism pathway may play an important role in the process of NAFLD-hepatocellular carcinoma. In addition, three glucose metabolism-related genes (SERPINE1, VCAN, and TFPI2) may be potential targets for the immunotherapy of patients with NAFLD-hepatocellular carcinoma.

## Data Availability

The data used to support the findings of this study were supplied by Peng Yao under license and so cannot be made freely available. Requests for access to these data should be made to Peng Yao, email: yp113065@outlook.com.

## References

[1] Powell E. E., Wong V. W. S., Rinella M. (2021). Non-alcoholic fatty liver disease. *The Lancet*.

[2] Neuschwander-Tetri B. A. (2017). Non-alcoholic fatty liver disease. *BMC Medicine*.

[3] Maurice J., Manousou P. (2018). Non-alcoholic fatty liver disease. *Clinical Medicine*.

[4] Engin A. (2017). Non-alcoholic fatty liver disease. *Advances in Experimental Medicine and Biology*.

[5] Santhekadur P. K., Kumar D. P., Sanyal A. J. (2018). Preclinical models of non-alcoholic fatty liver disease. *Journal of Hepatology*.

[6] Francque S. M., Marchesini G., Kautz A. (2021). Non-alcoholic fatty liver disease: a patient guideline. *JHEP Reports*.

[7] Ter Horst K. W., Serlie M. J. (2017). Fructose consumption, lipogenesis, and non-alcoholic fatty liver disease. *Nutrients*.

[8] Katsiki N., Mikhailidis D. P., Mantzoros C. S. (2016). Non-alcoholic fatty liver disease and dyslipidemia: an update. *Metabolism*.

[9] Shaunak M., Byrne C. D., Davis N., Afolabi P., Faust S. N., Davies J. H. (2021). Non-alcoholic fatty liver disease and childhood obesity. *Archives of Disease in Childhood*.

[10] Bedossa P. (2017). Pathology of non-alcoholic fatty liver disease. *Liver International*.

[11] Mayneris-Perxachs J., Cardellini M., Hoyles L. (2021). Iron status influences non-alcoholic fatty liver disease in obesity through the gut microbiome. *Microbiome*.

[12] Yazıcı D., Sezer H. (2017). Insulin resistance, obesity and lipotoxicity. *Advances in Experimental Medicine and Biology*.

[13] Li X., Shi Z., Zhu Y. (2020). Cyanidin-3-O-glucoside improves non-alcoholic fatty liver disease by promoting PINK1-mediated mitophagy in mice. *British Journal of Pharmacology*.

[14] Houttu V., Csader S., Nieuwdorp M., Holleboom A. G., Schwab U. (2021). Dietary interventions in patients with non-alcoholic fatty liver disease: a systematic review and meta-analysis. *Frontiers in Nutrition*.

[15] Choudhary N. S., Duseja A. (2021). Genetic and epigenetic disease modifiers: non-alcoholic fatty liver disease (NAFLD) and alcoholic liver disease (ALD). *Transl Gastroenterol Hepatol*.

[16] Hu B., Yu M., Ma X. (2022). IFN*α* potentiates anti-PD-1 efficacy by remodeling glucose metabolism in the hepatocellular carcinoma microenvironment. *Cancer Discovery*.

[17] Zheng Y. L., Li L., Jia Y. X. (2019). LINC01554-Mediated glucose metabolism reprogramming suppresses tumorigenicity in hepatocellular carcinoma via downregulating PKM2 expression and inhibiting akt/mTOR signaling pathway. *Theranostics*.

[18] Ren X., Chen X., Zhang X. (2021). Immune microenvironment and response in prostate cancer using large population cohorts. *Frontiers in Immunology*.

[19] Bence K. K., Birnbaum M. J. (2021). Metabolic drivers of non-alcoholic fatty liver disease. *Molecular Metabolism*.

[20] Hazlehurst J. M., Woods C., Marjot T., Cobbold J. F., Tomlinson J. W. (2016). Non-alcoholic fatty liver disease and diabetes. *Metabolism*.

[21] Jennison E., Patel J., Scorletti E., Byrne C. D. (2019). Diagnosis and management of non-alcoholic fatty liver disease. *Postgraduate Medical Journal*.

[22] Abenavoli L., Boccuto L., Federico A. (2019). Diet and non-alcoholic fatty liver disease: the mediterranean way. *International Journal of Environmental Research and Public Health*.

[23] Chen D., Wang Y., Lu R. (2020). E3 ligase ZFP91 inhibits Hepatocellular Carcinoma Metabolism Reprogramming by regulating PKM splicing. *Theranostics*.

[24] Liu Y., Zhang Y., Xiao B. (2021). MiR-103a promotes tumour growth and influences glucose metabolism in hepatocellular carcinoma. *Cell Death and Disease*.

[25] Shang R. Z., Qu S. B., Wang D. S. (2016). Reprogramming of glucose metabolism in hepatocellular carcinoma: progress and prospects. *World Journal of Gastroenterology*.

[26] Zhang Z., Tan X., Luo J., Yao H., Si Z., Tong J. S. (2020). The miR-30a-5p/CLCF1 axis regulates sorafenib resistance and aerobic glycolysis in hepatocellular carcinoma. *Cell Death and Disease*.

[27] Tan R., Zhang G., Liu R. (2021). Identification of early diagnostic and prognostic biomarkers via WGCNA in stomach adenocarcinoma. *Frontiers Oncology*.

[28] Li R., Hou S., Zou M., Ye K., Xiang L. (2021). miR-543 impairs cell proliferation, migration, and invasion in breast cancer by suppressing VCAN. *Biochemical and Biophysical Research Communications*.

[29] Bai K. H., He S. Y., Shu L. L. (2020). Identification of cancer stem cell characteristics in liver hepatocellular carcinoma by WGCNA analysis of transcriptome stemness index. *Cancer Medicine*.

[30] Li W., Han F., Fu M., Wang Z. (2020). High expression of VCAN is an independent predictor of poor prognosis in gastric cancer. *Journal of International Medical Research*.

[31] Zhao D., Qiao J., He H., Song J., Zhao S., Yu Y. (2020). TFPI2 suppresses breast cancer progression through inhibiting TWIST-integrin α5 pathway. *Molecular Medicine*.

[32] Li T., Gao X., Han L., Yu J., Li H. (2018). Identification of hub genes with prognostic values in gastric cancer by bioinformatics analysis. *World Journal of Surgical Oncology*.

[33] Jiang K., Liu H., Xie D., Xiao Q. (2019). Differentially expressed genes ASPN, COL1A1, FN1, VCAN and MUC5AC are potential prognostic biomarkers for gastric cancer. *Oncology Letters*.

[34] Behary J., Amorim N., Jiang X. T. (2021). Gut microbiota impact on the peripheral immune response in non-alcoholic fatty liver disease related hepatocellular carcinoma. *Nature Communications*.

[35] Zeng F., Zhang Y., Han X., Zeng M., Gao Y., Weng J. (2020). Predicting non-alcoholic fatty liver disease progression and immune deregulations by specific gene expression patterns. *Frontiers in Immunology*.

[36] Zhang X., Lu Z., Ren X. (2021). Genetic comprehension of organophosphate flame retardants, an emerging threat to prostate cancer. *Ecotoxicology and Environmental Safety*.

[37] Pei J. P., Zhang C. D., Yusupu M., Zhang C., Dai D. Q. (2021). Screening and validation of the hypoxia-related signature of evaluating tumor immune microenvironment and predicting prognosis in gastric cancer. *Frontiers in Immunology*.

[38] Sung J. Y., Cheong J. H. (2022). Machine learning predictor of immune checkpoint blockade response in gastric cancer. *Cancers*.

[39] Zhang X., Ren X., Zhang T. (2022). Comprehensive analysis of the association between human non-obstructive azoospermia and plasticisers via single-cell and traditional rna sequencing methods. *Exposure and Health*.

[40] Zhang X., Zhang T., Ren X., Chen X., Wang S., Qin C. (2021). Pyrethroids toxicity to male reproductive system and offspring as a function of oxidative stress induction: rodent studies. *Frontiers in Endocrinology*.

[41] Liu S., He B., Li H. (2022). Comprehensive analysis of emerging flame retardants, a risk factor to prostate cancer?. *Ecotoxicology and Environmental Safety*.

